# Salience and default networks predict borderline personality traits and affective symptoms: a dynamic functional connectivity analysis

**DOI:** 10.3389/fnhum.2025.1589440

**Published:** 2025-07-01

**Authors:** Alessandro Grecucci, Miriam Langerbeck, Richard Bakiaj, Parisa Ahmadi Ghomroudi, Davide Rivolta, Xiaoping Yi, Irene Messina

**Affiliations:** ^1^Department of Psychology and Cognitive Sciences (DiPSCo), University of Trento, Trento, Italy; ^2^Faculty of Psychology and Neuroscience (FPN), Maastricht University, Maastricht, Netherlands; ^3^Department of Education, Psychology and Communication, University of Bari Aldo Moro, Bari, Italy; ^4^Department of Radiology, Chongqing University Three Gorges Hospital, Chongqing University, Chongqing, China; ^5^Clinical Research Center (CRC), Medical Pathology Center (MPC), Cancer Early Detection and Treatment Center (CEDTC) and Translational Medicine Research Center (TMRC), Chongqing University Three Gorges Hospital, Chongqing University, Chongqing, China; ^6^School of Medicine, Chongqing University, Chongqing, China; ^7^Department of Human and Social Sciences, Mercatorum University, Rome, Italy

**Keywords:** borderline personality disorder, personality traits, unsupervised machine learning, default mode network, salience network

## Abstract

**Introduction:**

Borderline personality disorder (BPD) is one of the most frequently diagnosed disorders in psychiatric settings. Beyond the categorical diagnosis, borderline personality traits (BPT) are common in the general population and vary along a continuum from mild to severe. While prior research has reported functional connectivity alterations in the default mode network (DMN), the salience network (SN), and the central-executive network (CEN) in patients with BPD, the impairment of these networks in subclinical BPT remain underexplored. To fill this gap, this study aims to investigate dynamic functional connectivity alterations associated with BPT in a subclinical population. We expect to find abnormal connectivity inside the DMN, the SN and in regions ascribed to mentalization processes associated with BPT. We also expect these networks to be associated with psychological symptoms experienced by borderline patients such as impulsivity and anger issues, as well as lack of self-control and neuroticism among others.

**Method:**

An unsupervised machine learning method known as Group-ICA, was applied to resting state fMRI images of 200 individuals to predict BPT from the temporal variability of independent macro networks.

**Results:**

Results indicated abnormal dynamic functional connectivity inside the SN including areas implicated in emotional reactivity and sensitivity, and in a network that partially overlaps with the DMN, including regions involved in social cognition and mind reading. Specifically, the higher the BPT, the higher the temporal variability inside the SN, and the lower the temporal variability in a network that includes DMN and mentalization regions. Notably, the BOLD variability of the SN correlated with neuroticism, anger problems, lack of self- control, and distorted inner dialogue, all symptoms displayed by individuals with borderline personality.

**Discussion:**

These findings indicate that abnormalities in resting state networks are visible in subclinical populations with varying degrees of borderline traits, with impaired DMN and SN. These insights may pave the way for designing interventions to prevent the development of the full disorder.

## 1 Introduction

Borderline personality traits (BPT) are distributed continuously across the population, existing along a spectrum of psychopathological severity, with borderline personality disorder (BPD) representing the most extreme manifestation. BPD is formally recognized as a psychiatric disorder in the Diagnostic and Statistical Manual of Mental Disorders (DSM-5), characterized by pervasive patterns of emotional dysregulation, impulse control issues, unstable interpersonal relationships, distorted inner dialogue, and an inconsistent self-image (American Psychiatric Association, 2013). These patterns often manifest through impulsive aggressive behavior, unstable relationships, self-harming actions, and chronic suicidal tendencies (Mendez-Miller et al., 2022; Dadomo et al., 2016). According to dimensional models of personality disorders (Cuthbert, 2014; Hörz-Sagstetter et al., 2021), subclinical BPT and their neural correlates are likely qualitatively similar but quantitatively less severe than those observed in clinical cases. Therefore, there may be intermediate phenotypic forms of BPT in the general population that do not reach the threshold for clinical diagnosis, but still deserve attention due to their potential role as important predictors for the development of BPD (Wright et al., 2015; Bozzatello et al., 2021; De Panfilis et al., 2019). The identification of neural biomarkers that capture subclinical BPT could significantly clarify the mechanisms underlying the development of the full disorder. Traditional neuroscientific models of BPD attribute impulsivity and poor emotional regulation to prefrontal-limbic dysregulation (Perez-Rodriguez et al., 2018; Herpertz et al., 2018; Krause-Utz et al., 2014). These models describe BPD dysfunction in terms of amygdala hyperreactivity (Donegan et al., 2003), combined with impaired recruitment of the cognitive executive network (CEN), including the dorsolateral and dorsomedial prefrontal cortex (dlPFC, dmPFC), the dorsal anterior cingulate cortex (dACC), and the ventrolateral prefrontal cortex (vlPFC). This impaired recruitment is conceptualized as a failure of “top-down” regulation, where higher-order cortical regions fail to exert control over the emotional responses generated by subcortical limbic structures (Taylor and Liberzon, 2007). However, other models of BPD do exist. Beyond the top-down control model of BPD, a new line of research has highlighted the role of three resting state macronetworks: the default mode network (DMN), the salience network (SN), and the central executive network (CEN), collectively known as the triple network model. Recent studies have confirmed the triple network model by showing abnormal functional connectivity in BPD patients when compared to healthy controls inside the SN, the DMN and the CEN (Doll et al., 2013; Ruocco and Carcone, 2016; Xiao et al., 2024). These findings have also been supported by research on structural connectivity in BPT (Quattrini et al., 2022; Langerbeck et al., 2023). The abnormal connectivity inside the DMN in individuals with borderline personality disorder (BPD), which includes anterior and posterior medial regions such as the medial frontal cortex and the precunesus, was related with an exaggerated focus on autobiographical or self-referential information (Beeney et al., 2016; Aguilar-Ortiz et al., 2020), but also with emotion dysregulation, impulsivity (Langerbeck et al., 2023; Grecucci et al., 2022, 2023), and a disrupted ability to differentiate between self and others (Sharp et al., 2013; Beeney et al., 2016). More precisely, the brain regions of posterior cingulate cortex (PCC)/precuneus, and the angular gyrus (AG) are affected, which are closely linked to social cognition tasks (Schilbach et al., 2008; Schurz et al., 2021) and self-related functions (Buckner and Carroll, 2007; Messina et al., 2016). These findings have been interpreted as a failure of mentalization and social cognition in BPD (Schurz et al., 2024). Mentalization refers to the ability to understand oneself and others by interpreting social behavior in terms of subjective mental states and processes, such as thoughts, feelings, and beliefs. This enables an understanding of others’ actions based on their likely inner experiences (Fonagy et al., 2011).

While DMN alterations reflect emotional dysregulation, SN changes may underlie emotional reactivity. The SN includes both the anterior and posterior insula (AI, PI) and the anterior cingulate cortex (ACC), and may also contribute to BPD due to its role in threat detection, anxiety symptoms (Baggio et al., 2023; under review), and for its pivotal role in switching between the CEN and the DMN (Goulden et al., 2014). Indeed, BPD patients seem to have difficulties in switching attention from baseline resting-state to external, task-related demands (Doll et al., 2013). In addition, the SN plays a crucial role in processing emotions and interoception (Chong et al., 2017) and is associated with individual differences in the domain of socio-emotional sensitivity (Toller et al., 2018). Consistently, the higher activation of SN observed in BPD is explained as the excessive emotional reactivity which characterize BPD (Denny et al., 2018).

For what concerns the CEN, a network of brain regions including the dorsolateral prefrontal cortex (dlPFC) and the lateral posterior parietal cortex (PPC), this network plays a key role in executive control during goal-oriented actions and is essential for holding and processing information in working memory when engaging in tasks that demand attention (Menon, 2011; Quattrini et al., 2022). This network may be related with lack of control and modulation over impulses and emotions, BPD suffer from.

An open question concerns the validity of the available neuroscientific models for describing the subclinical borderline personality in the form of borderline personality traits (BPT). Two alternative hypotheses emerge in this context. The first hypothesis suggests that the neural correlates of subclinical BPT are qualitatively similar to those observed in BPD, differing only in their quantitative expression. The second hypothesis posits that the continuum between subclinical BPT and BPD applies only to certain networks and the related psychological processes, while others may exhibit qualitative differences (Langerbeck et al., 2023). This second version was partially confirmed by morphometric studies conducted by Langerbeck et al. (2023) who showed that only the DMN was affected in BPT but not the SN or the CEN (Langerbeck et al., 2023). However, we still do not know whether this applies to functional resting state connectivity metrics. In other words, it remains unclear whether the same functional brain networks associated with BPD are similarly altered in individuals with BPT, or whether these alterations are more restricted. One possibility, in line with recent structural findings by Langerbeck et al. (2023), is that functional alterations in BPT are confined to the DMN, without involving the other networks (SN and CEN). In the current study we test this hypothesis confirmatory. The hypothesis aligns with the notion that functional disruptions in BPT may differ qualitatively to BPD but only in certain networks. However, it is also possible that functional changes in BPT extend beyond the DMN and include the SN and CEN, resembling the broader network dysfunctions observed in BPD. Given the limited prior research on functional alterations in BPT, we are exploring this possibility in a more open-ended way to see if a more widespread pattern of dysfunction is already present at a subclinical level. This possibility would be consistent with the assumption that BPT do not differ qualitatively from BPD, but only quantitatively.

To investigate this, the first aim of the present study was to test the dynamic functional connectivity of all macro-networks for predicting BPT in a large subclinical population. We hypothesize that altered connectivity will be detected inside the DMN. To investigate the possibility of our exploratory hypothesis we also looked at the SN, and the CEN. We excluded the possibility of the involvement of other well-known macro-networks (visual, sensorimotor, attentional, linguistic, cerebellar). Of note, the previous attempts to study the brain alterations in BPT did not relate brain alterations to specific affective symptoms that characterize borderline personalities. Previous studies clearly showed that the networks that differ from controls are also related to affective and cognitive symptoms in both adults (Grecucci et al., 2022, 2023; Sorella et al., 2019) and children (Xiao et al., 2024) diagnosed with BPD. An interesting question is whether the same relationship between brain changes and affective symptoms observed in fully diagnosed BPD can be found in individuals with borderline personality traits (BPD). Previous studies have shown a relationship between BPD and other personality traits (Hopwood and Zanarini, 2010), suggesting that at least a few similar patterns may occur in individuals with subclinical traits. Therefore, the second aim of this study is to test the hypothesis that at least some of these three networks may be associated with psychological dysfunctions as measured by the dedicated questionnaires. Specifically, we hypothesize that the higher the alteration in connectivity inside those networks, the higher the affective (anger, impulsivity), the cognitive (inner dialogue, self-control), and the personality symptoms (neuroticism).

To test our hypotheses, resting-state networks were identified via an unsupervised machine learning method known as dynamic independent component analysis (dICA) applied to resting state fMRI images. Recent evidence suggests that connectivity patterns are not static, but change over time (Calhoun et al., 2014; Chang and Glover, 2009). In line with recent research (Preti et al., 2017), our current study expands on previous findings by considering the variability of the temporal dynamics of ICA-based networks. The study of the dynamic fluctuations in functional connectivity (FC) patterns during fMRI scans, referred to as dynamic FC (dFC), has become increasingly prominent (Preti et al., 2017; Cavanna et al., 2018; Shunkai et al., 2022). This method has demonstrated potential for predicting changes in brain functions in both normal conditions (Long et al., 2023a, 2023b; Omidvarnia et al., 2021; Chen et al., 2017) and pathological states (Long et al., 2020; Long et al., 2023a, 2023b; Zhang et al., 2016).

## 2 Methods

### 2.1 Participants

For this study, we used data from the Max Planck Institute sample (MPI-S) dataset (OpenNeuro database, accession number ds000221), which contains behavioral as well as structural and functional neuroimaging data from 321 German-speaking, healthy subjects (Babayan et al., 2018). Inclusion criteria were completion of the questionnaires, and medical eligibility for magnetic resonance sessions. Exclusion criteria included the following: history of neurological or psychiatric diagnosis [controlled with the SCID-I (Wittchen et al., 1995)], drug use, medication such as cortisol, beta blockers, chemotherapeutic or psychopharmacological drugs. For this study, we selected participants according to age (20–69), availability of structural T1-weighted images and 15-min eyes-open resting state data and availability of specific questionnaire scales. The final sample included 200 subjects (*M* = 95, *F* = 105), mean age of 32.43 years (SD = 13.92). In this study we used the Personality Style and Disorder Inventory (PSSI) to detect the level of BPT, and the following questionnaire to psychologically characterize the neural findings: the Varieties of Inner Speech Questionnaire (VISQ, McCarthy-Jones and Fernyhough, 2011), the State-Trait Anger Expression Inventor (STAXI, Spielberger, 1996), the Big-Five Personality (NEO-PI, Costa and McCrae, 1992), and the Self-Control Scale (Tangney et al., 2004).

### 2.2 fMRI data

Neuroimaging data were acquired on a 3 T Siemens Magnetom Verio Scanner. For our analyses, we took into account T1-weighted images, acquired using a MP2RAGE sequence (TR = 5,000 ms, TE = 2.92 ms, TI1 = 700 ms, TI2 = 2,500 ms, flip angle 1 = 4, flip angle 2 = 5, voxel size = 1.0 mm isotropic, duration = 8.22 min), and the 15-min resting-state data (voxel size = 2.3 mm isotropic, FOV = 202,202 mm^2^, imaging matrix = 88 88, 64 slices with 2.3 mm thickness, TR = 1,400 ms, TE = 39.4 ms, flip angle = 69, echo spacing = 0.67 ms, bandwidth = 1776 Hz/Px, partial Fourier 7/8, no pre-scan normalization, multiband acceleration factor = 4, 657 volumes, duration = 15 min 30 s).

### 2.3 Preprocessing

Resting state fMRI data pre-processing was conducted using CONN (version 2022), SPM12, and the MATLAB Toolbox (version 2021b). The default pre-processing pipeline in CONN and SPM12’s default parameters were used. The pre-processing steps involved functional realignment and unwarping, followed by translation and centering. Then, Conservative functional outlier detection was performed. The functional data were segmented and normalized to a 2 mm resolution, while structural data underwent translation, centering, segmentation, and normalization to a 2 mm resolution. Finally, spatial smoothing of both functional and structural data was applied using an 8 mm Gaussian kernel. Next, denoising was performed to remove confounding variables and artifacts from the BOLD signal. Artifacts stemmed from white matter, cerebrospinal fluid (CSF) signals, parameters and outliers defined during the pre-processing step, and estimated motion parameters. These factors were included as covariates in a regression model. Finally, the time series was subjected to temporal bandpass filtering within the 0.0008 Hz to infinity range.

### 2.4 Group-ICA

**Figure 1:**
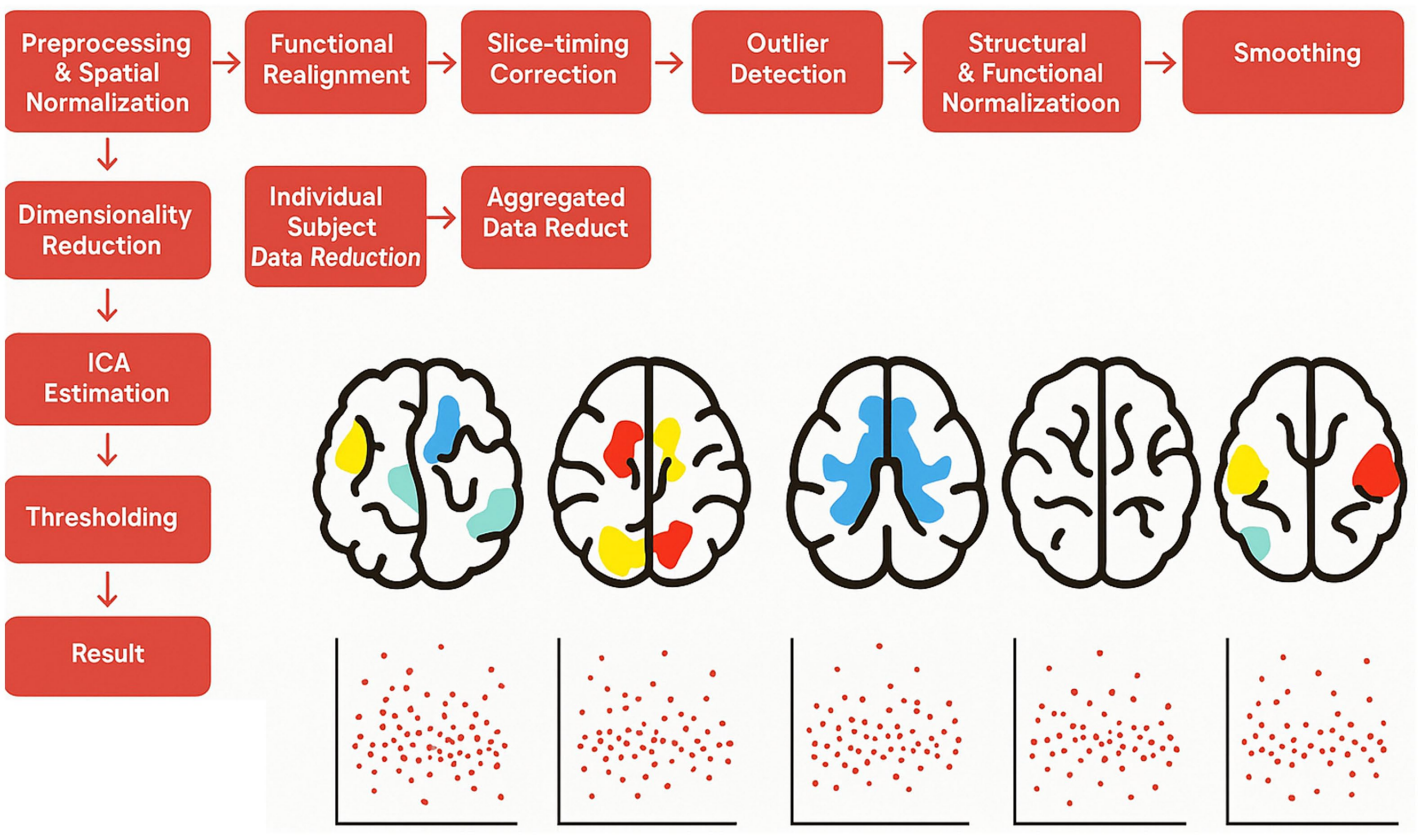
Methods CONN. Schematic diagram of the Group ICA: the resting-state data was first preprocessed, followed by the extraction of 20 independent components using, Group ICA.

Connectivity analysis was conducted using the data-driven group-independent component approach (group-ICA) in CONN. The group-ICA process included several steps: first, variance normalization (pre-conditioning); followed by temporal concatenation of the BOLD signal; then, group-level dimensionality reduction; next, fast-ICA for spatial component estimation; and finally, back-projection for individual spatial estimation. Following default parameters, 20 independent components were extracted (Calhoun et al., 2001), in line with previous studies (Zanella et al., 2022; Ghomroudi et al., 2024). Each IC was visually inspected and compared with CONN’s network atlas using a spatial match-to-template function to distinguish noise components from resting-state networks. The temporal variability and frequency of each IC were then determined by calculating the standard deviation of the BOLD time series. To control the risk of Type I errors, a cluster-size-based false discovery rate (FDR) correction was applied, with significance set at *p* < 0.05 and a voxel threshold of *p* < 0.001 for each analysis. To determine which of the 20 identified ICs were predictive of the BPT score, backward stepwise regression analyses were conducted using the regression module of JASP (Version 0.16.2; JASP Team, 2022). The variability and frequency of the ICs, along with age and gender, were included as predictors, with the BPT score as the dependent variable (see Figure 1).

## 3 Results

### 3.1 Resting-state analysis

**Figure 2:**
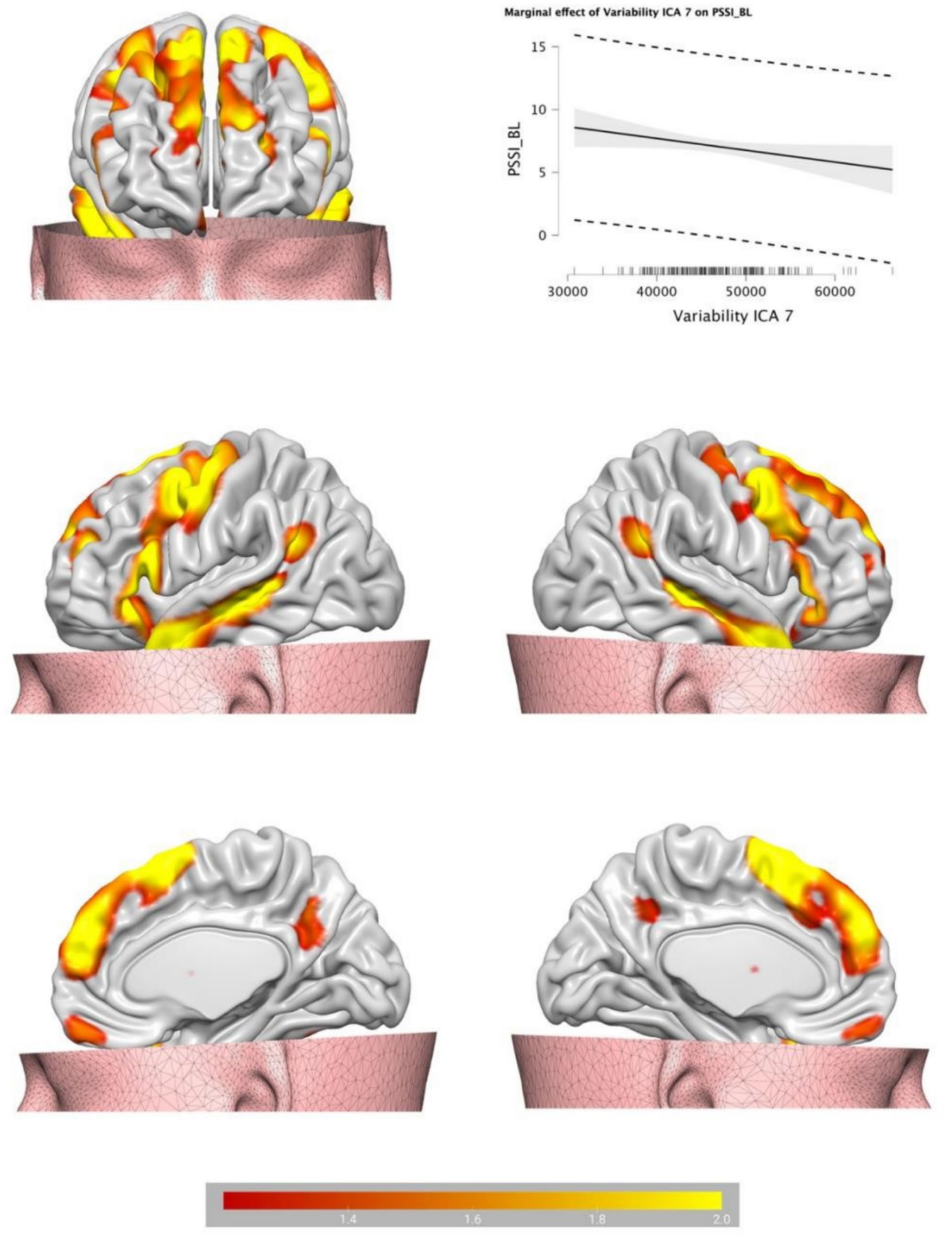
Brain plots of the BOLD temporal variability inside the IC7, partially identified as default mode network.

**Figure 3:**
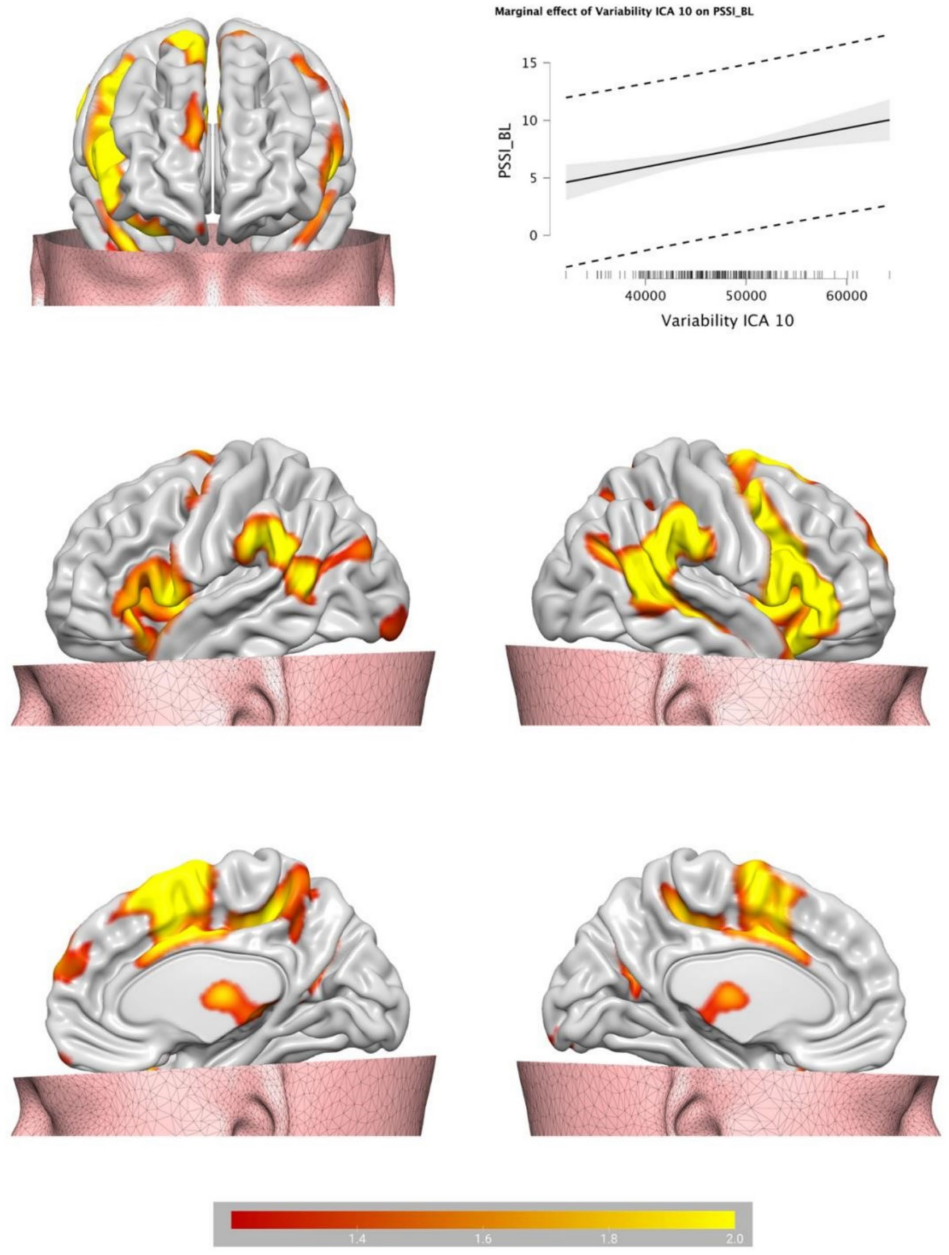
Brain plots of the BOLD temporal variability inside the IC10, identified as salience network.

Backward Stepwise Regression analysis returned a significant profit model (*F* = 4.717, *p* < 0.001). The BOLD variability of IC7 (*β* = −0.141, *p* = 0.046) and of IC 10 (*β* = 0.237, *p* < 0.001) were predictive of BPT. See Table 1 for IC 7 and IC 10 encompass a cluster of regions at cluster statistical significance level of *p* < (pFDR corrected) and at the voxel significant level *p* < (pFDR corrected). IC7 includes frontal areas (e.g., middle frontal gyrus), temporal areas (e.g., middle temporal gyrus), the hippocampus, and largely overlaps with the DMN, and has a negative relationship with BPT, the lower the variability, the higher the BPT. IC10 includes the insula, the cingulate, the thalamus, the amygdala, the inferior frontal regions among others, and overlaps with the SN, and has a positive relationship with BPT, the higher the variability, the higher the BPT. See Table 2 for IC7, and Table 3 for IC10 (see Figures 2, 3).

**Table 1:** Result of backward regression.

Variable	*β*	SE	*t*	*p*	95% CI
Variability ICA 7	−9.413 × 10^−5^	4.685 × 10^−5^	−2.009	0.046	[−0.00019, −0.000002]
Variability ICA 10	1.689 × 10^−4^	5.004 × 10^−5^	3.375	<0.001	[0.00007, 0.00027]

SE, standard error; CI, confidence interval.

**Table 2:** IC7.

ROI	Voxels	Peak statistics	MNI coordinates (mm)
100% of aMTG r (middle temporal gyrus, ant R)	409	27	(+58, −2, −24)
100% of aMTG l (middle temporal gyrus, ant LL)	449	27.8	(−58, −4, −22)
100% of aITG l (inferior temporal gyrus, ant LL)	335	20.8	(−48, −6, −40)
98% of pMTG r (middle temporal gyrus, post R)	1,330	28.5	(+60, −22, −12)
98% of caudate r	511	22.4	(+14, +10, +10)
98% of caudate l	527	22.7	(−12, +10, +10)
98% of aITG r (inferior temporal gyrus, ant R)	321	21.4	(+46, −2, −40)
93% of SFG l (superior frontal gyrus L)	2,631	50.5	(−14, +20, +56)
93% of pMTG l (middle temporal gyrus, post L)	1,282	28.5	(−60, −26, −10)
90% of MidFG r (middle frontal gyrus R)	2,479	19.8	(+40, +18, +44)
89% of SFG r (superior frontal gyrus R)	2,398	35.4	(+14, +20, +56)
88% of PaCiG l (paracingulate gyrus L)	1,154	35.8	(−6, +36, +24)
88% of IFG oper l (inferior frontal gyrus, pars opercularis L)	672	20.1	(−50, +16, +16)
87% of IFG tri r (inferior frontal gyrus, pars triangularis R)	482	18.6	(+52, +28, +8)
86% of TOFusC r (temporal occipital fusiform cortex R)	703	15.6	(+34, −50, −16)
86% of PaCiG r (paracingulate gyrus R)	1,164	20.5	(+6, +36, +26)
84% of OFusG r (occipital fusiform gyrus R)	749	13.6	(+28, −74, −12)
84% of OFusG l (occipital fusiform gyrus L)	779	8.8	(−28, −76, −14)
83% of MedFC (frontal medial cortex)	817	15.6	(+0, +44, −20)
83% of AG r (angular gyrus R)	1,226	18.3	(+52, −52, +30)
80% of hippocampus r	555	10.7	(+28, −18, −16)
78% of Cereb6 r (cerebelum 6 R)	1,204	19.7	(+24, −62, −24)
78% of AG l (angular gyrus L)	740	16.7	(−50, −56, +28)
76% of pPaHC r (parahippocampal gyrus, post R)	241	10.6	(+24, −30, −18)
73% of pSTG l (superior temporal gyrus, post L)	286	25.8	(−62, −28, +2)
73% of PreCG l (precentral gyrus L)	3,202	30.1	(−36, −10, +52)
72% of PreCG r (precentral gyrus R)	3,070	17.4	(+38, −10, +52)
71% of MidFG l (middle frontal gyrus L)	2,087	31.3	(−38, +14, +44)
70% of TP l (temporal pole L)	1,654	36.3	(−44, +12, −30)
69% of TP r (temporal pole R)	1,633	37.3	(+42, +14, −32)
65% of IFG tri l (inferior frontal gyrus, pars triangularis L)	421	22.6	(−52, +26, +6)
65% of accumbens l	70	15.6	(−10, +14, −6)
62% of aSTG r (superior temporal gyrus, ant R)	171	25.1	(+56, −2, −14)
61% of hippocampus l	462	9.5	(−26, −20, −16)
61% of Cereb1 l (cerebelum crus1 L)	1,397	14.9	(−32, −72, −28)
61% of aSTG l (superior temporal gyrus, ant L)	170	24.4	(−54, −4, −12)
60% of pSTG r (superior temporal gyrus, post R)	249	26.6	(+58, −24, −2)

**Table 3:** IC10.

ROI	Voxels	Peak statistics	MNI coordinates (mm)
100% of thalamus r	1,269	22.1	(+10, −18, +6)
100% of thalamus l	1,361	18.2	(−10, −20, +6)
100% of SMA r (juxtapositional lobule cortex-formerly supplementary motor cortex-R)	714	36.5	(+6, −2, +58)
100% of SMA L (juxtapositional lobule cortex-formerly supplementary motor cortex-L)	643	28.5	(−6, −2, +56)
100% of IFG tri r (inferior frontal gyrus, pars triangularis R)	555	40.8	(+52, +28, +8)
100% of IFG oper r (inferior frontal gyrus, pars opercularis R)	686	33.9	(+52, +16, +16)
100% of IFG oper l (inferior frontal gyrus, pars opercularis L)	766	23.8	(−50, +14, +16)
100% of ICC l (intracalcarine cortex L)	640	15.3	(−10, −76, +8)
100% of FO r (frontal operculum cortex R)	313	34.6	(+42, +18, +4)
100% of FO l (frontal operculum cortex L)	355	23.7	(−40, +18, +4)
100% of aSMG r (supramarginal gyrus, ant R)	801	26.9	(+58, −28, +38)
100% of Ver8 (vermis 8)	240	13.9	(+2, −64, −34)
99% of Ver9 (vermis 9)	164	17.1	(+0, −54, −34)
99% of pallidum r	266	16.1	(+20, −4, −2)
99% of pallidum l	299	15.3	(−20, −6, −2)
98% of Cereb6 l (cerebelum 6 L)	1,269	23.1	(−22, −58, −24)
97% of Ver45 (vermis 4 5)	607	15.1	(+2, −52, −6)
96% of Ver6 (vermis 6)	322	14	(+2, −66, −16)
96% of ICC r (intracalcarine cortex R)	725	11.7	(+12, −74, +8)
95% of TP r (temporal pole R)	2,257	26.4	(+40, +14, −30)
95% of TOFusC l (temporal occipital fusiform cortex L)	619	13.4	(−34, −54, −16)
94% of SPL r (superior parietal lobule R)	1,382	17.1	(+28, −48, +58)
94% of IC l (insular cortex L)	1,259	23.5	(−36, +2, +0)
93% of toMTG r (middle temporal gyrus, temporooccipital part R)	1,077	29	(+58, −50, +2)
90% of IC r (insular cortex R)	1,208	26.2	(+38, +4, −2)
90% of Cereb45 r (cerebelum 4 5 R)	550	12.6	(+16, −46, −18)
89% of PO r (parietal operculum cortex R)	479	28.1	(+50, −28, +22)
88% of pSMG r (supramarginal gyrus, post R)	1,092	32.5	(+56, −40, +32)
88% of Cereb8 l (cerebelum 8 L)	1,642	26.3	(−24, −56, −48)
86% of Cereb9 l (cerebelum 9 L)	734	17.9	(−12, −50, −46)
86% of Cereb45 l (cerebelum 4 5 L)	775	13.1	(−14, −46, −16)
84% of IFG tri l (inferior frontal gyrus, pars triangularis L)	543	20.7	(−50, +28, +8)
83% of Ver7 (vermis 7)	161	14.4	(+0, −70, −26)
83% of FOrb r (frontal orbital cortex R)	1,193	37.7	(+32, +24, −16)
82% of putamen r	656	15.5	(+26, +0, +2)
82% of pPaHC r (parahippocampal gyrus, post R)	260	15.5	(+24, −32, −16)
80% of precuneous (precuneous cortex)	4,475	21	(+2, −58, +36)
79% of aSMG l (supramarginal gyrus, ant L)	750	25.3	(−58, −32, +34)
78% of SCC l (supracalcarine cortex L)	57	17.2	(−12, −66, +16)
77% of TP l (temporal pole L)	1,821	22.4	(−44, +12, −28)
77% of pTFusC l (temporal fusiform cortex, post L)	663	14.6	(−36, −34, −22)
77% of PO l (parietal operculum cortex L)	435	24.5	(−50, −32, +22)
76% of TOFusC r (temporal occipital fusiform cortex R)	619	15.1	(+36, −48, −18)
76% of Cereb10 l (cerebelum 10 L)	113	13.3	(−22, −36, −42)
75% of toMTG l (middle temporal gyrus, temporooccipital part L)	651	18.6	(−56, −54, +4)
73% of amygdala r	248	17.8	(+22, −4, −16)
71% of SFG r (superior frontal gyrus R)	1,906	36.3	(+12, +16, +58)
70% of PreCG l (precentral gyrus L)	3,038	24.6	(−36, −8, +46)
69% of Cereb1 l (cerebelum crus1 L)	1,591	23.9	(−32, −68, −28)
67% of SCC r (supracalcarine cortex R)	96	15.1	(+12, −64, +16)
67% of pPaHC l (parahippocampal gyrus, post L)	263	13.7	(−22, −34, −14)
67% of CO l (central opercular cortex L)	660	24.7	(−46, −6, +12)
67% of aITG r (inferior temporal gyrus, ant R)	219	12.7	(+46, +0, −40)
66% of OP l (occipital pole L)	1,734	17.2	(−20, −98, +0)
66% of Cereb6 r (cerebelum 6 R)	1,028	15	(+24, −56, −24)
65% of AC (cingulate gyrus, ant)	1,697	24.7	(+0, +10, +32)
64% of pTFusC r (temporal fusiform cortex, post R)	459	15	(+36, −28, −24)
63% of putamen l	547	11.9	(−26, −4, +4)
62% of FOrb l (frontal orbital cortex L)	1,045	23	(−36, +22, −14)
61% of pSMG l (supramarginal gyrus, post L)	653	24.6	(−58, −46, +24)
61% of PreCG r (precentral gyrus R)	2,627	34.9	(+36, −8, +46)
61% of amygdala l	201	11.2	(−22, −4, −16)
61% of AG r (angular gyrus R)	892	29	(+54, −52, +24)
60% of sLOC r (lateral occipital cortex, superior division R)	2,910	21.1	(+32, −70, +40)

### 3.2 Correlations

IC10 was significantly correlated with the inner dialogue scale—other voice subscale (*ρ* = 0.177 *p* = 0.012); Self-control scale (*ρ* = − 0.146 *p* = 0.039), NEO—neuroticism subscale (*ρ* = 0.0218 *p* = 0.002), UPSS negative urgency subscale (*ρ* = 0.216 *p* = 0.002), Staxi anger-trait (*ρ* = 0.152 *p* = 0.032), Staxi anger-out (*ρ* = 0.142 p = 0.046) and Staxi anger-control (*ρ* = − 0.180 *p* = 0.011).

**Figure 4:**
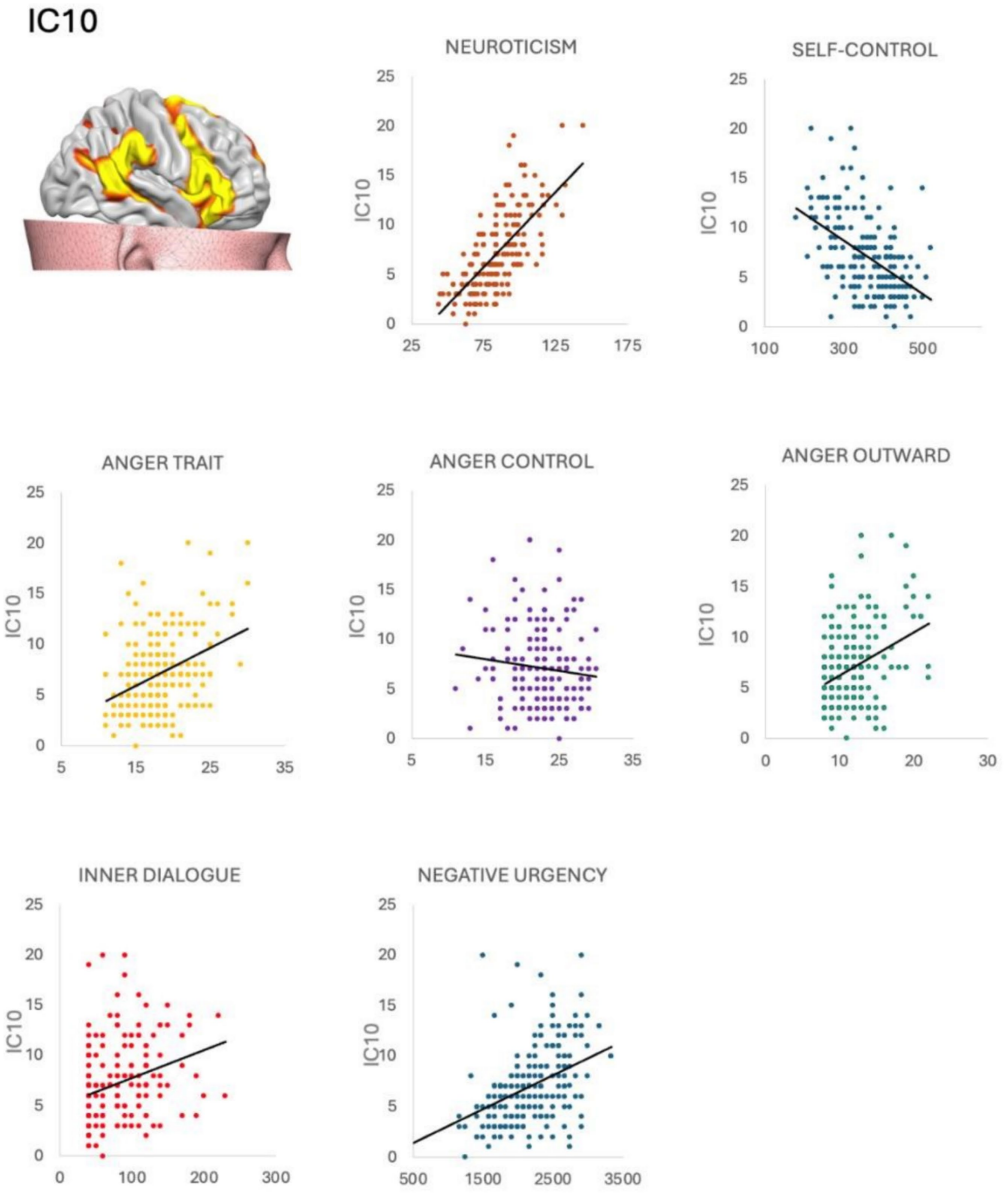
Correlations between salience network and psychological questionnaires. The salience network (IC10) was positively correlated with impulsivity urgency, anger trait, neuroticism, and negatively correlated with anger control, distorted inner dialogue, and self-control. Neuroticism, anger trait, anger outward, inner dialogue and negative urgency were positively correlated with BPT. Self control and anger control were negatively correlated with BPT.

IC7 was not correlated with any questionnaire considered (*p* > 0.05) (see Figure 4).

## 4 Discussion

In the present study, we tested the competing hypotheses of functional alterations within the DMN and functional alteration within all three networks, that characterize individuals with subclinical BPT. The temporal dynamics of functional connectivity in resting-state fMRI data from 200 participants was considered to this aim. Specific resting-state macro networks were identified using dynamic independent component analysis (dICA). An unsupervised machine learning approach and regression analysis were then conducted to examine the associations between these networks and borderline traits. Our analyses revealed two significant patterns of connectivity associated with the severity of BPT. IC10, which was identified as salience network, and IC7, which partially overlaps with the DMN, were both associated with BPT severity, with IC10 showing the strongest statistical association. Of note, the higher the BPT the higher the IC10 and the lower the IC7. Interestingly, we did not find clear involvement of the CEN in individuals with BPT. This contrasts with our confirmatory hypothesis but aligns with our exploratory hypothesis, based on the structural findings of Langerbeck et al. (2023), which suggested that only some networks may be affected in BPT. One possibility is that CEN alterations are only visible for BPD but not for subclinical BPT. In other words, alterations in cognitive functions may be minimal at subclinical level and become visible only when the trait overcomes the diagnostic threshold. Whereas deficits in the DMN and the SN may be visible at earlies stages (subclinical BPT). These findings expand our previous knowledge on the structural alterations of the DMN in BPT, by showing that both the DMN and SN are functionally affected in BPT, but not the CEN. In the next sections we describe the results in detail.

### 4.1 The altered salience network

The strongest pattern of brain connectivity associated to BPT showed increased BOLD temporal variability in a set of areas belonging to the SN. These areas included subcortical regions (the amygdala, the putamen and the thalamus), the dACC, the insula, the AG/temporo-parietal junction (TPJ) and the inferior frontal gyrus (IFG). The salience network plays a critical role in identifying and processing emotionally relevant stimuli (Riedel et al., 2018; Seeley, 2019), switching between internal and external attention (Goulden et al., 2014), and modulating emotional responses (Viviani, 2013; Zanella et al., 2022). Increased connectivity in this network has been previously linked to excessive emotional reactivity (Toller et al., 2018). Consistent with these findings, increased SN connectivity has been previously found in BPD (Beeney et al., 2016; Aguilar-Ortiz et al., 2020), supporting the clinical observation that individuals with BPD often exhibit heightened emotional sensitivity and reactivity—a core feature of borderline pathology (American Psychiatric Association, 2013). The SN is also crucial for facilitating the switch between executive tasks (CEN) and resting state (DMN) activity (Goulden et al., 2014). The interaction between the SN and the IFG is particularly relevant in this context, given the IFG’s role in regulating emotional responses (Messina et al., 2015; Morawetz et al., 2017). Emotional interference studies have reported that the anterior insula and IFG are involved in emotion-related response inhibition (Hung et al., 2018; Puiu et al., 2020). Consistently, functional connectivity between the insula and the IFG has been shown to be negatively correlated with the efficacy of emotion regulation strategies (Li et al., 2021). Building on this prior research, sensitivity and reactivity, and their impact on maintaining executive control in the presence of emotional responses, seems to be distinctive feature of BPD. In the present study, this characteristic appears to be observable even in subclinical presentations of BPT. This interpretation of our data is further supported by the observed correlations between alterations in the SN and self-reported scores on various psychological dimensions. Our results indicate that greater BOLD temporal variability within the SN is associated with higher levels of trait anger and neuroticism, anger expression, negative urgency, and inner dialogue, while being inversely related to anger regulation and self-control. These findings suggest a coherent maladaptive emotional profile characterized by increased emotional sensitivity and difficulty in the inhibition of negative emotions. This supports the idea that alterations in SN functioning are not exclusive to clinical populations but extend along a continuum of borderline traits, reinforcing the dimensional nature of personality disorders.

### 4.2 The default mode network

While alterations in the SN reflect increased emotional reactivity, changes in the DMN may relate to difficulties in emotion regulation. Consistent with this idea, our study identified a second pattern of brain connectivity related to BPT, characterized by reduced temporal BOLD variability. This reduction was observed in regions partially corresponding to the anterior and temporal nodes of the DMN, particularly the dmPFC and the temporo-parietal regions. The DMN is active during periods of rest and includes two main nodes: the posterior cingulate cortex (PCC) and the medial prefrontal cortex. Other involved regions, such as the hippocampus and cingulate gyrus, contribute to processing semantic memories and internal thought. Meanwhile, the medial prefrontal cortex is associated with social cognition, self-reflection, and emotion regulation (Menon, 2011). IC7 mainly included fronto-medial regions of the DMN and as such are probably associated with altered self-representation, emotion regulation and self-reflection. Unfortunately, we did not measure these psychological processes, so these considerations remain speculative for the moment. Future studies may want to further explore these issues. The more temporo-parietal part of IC7 included large areas of the superior and middle temporal gyri, extending into the AG/TPJ. These regions partially overlap with the DMN, but also with the so called “mentalizing network,” in which the dmPFC and the AG/TPJ serve as the primary components, facilitating the ability to represent mental states based on various types of social information with differing complexities (Molenberghs et al., 2016; Fehlbaum et al., 2022). Within this network, the dmPFC plays a pivotal role in integrating higher-order information from others and external sources into the self across cognitive domains (Martin et al., 2017; Arioli et al., 2021). The dmPFC works in tandem with the AG/TPJ, which is more involved in retrieving perceptual knowledge about others and reasoning from their perspectives (Van Overwalle, 2009; Golec-Staśkiewicz et al., 2022). Notably, the IFG and temporal regions identified in this study also contribute to mentalization and social cognition, with the IFG playing a role in controlled retrieval, and the temporal areas involved in the storage of semantic information critical for social cognition (Schurz et al., 2014; Diveica et al., 2021). This patterns of reduced connectivity within the mentalizing network highlight key features of borderline personality, particularly affecting the integration and processing of complex social and self-referential information, which are central to mentalization and social cognition. This finding aligns with recent neurobiological models that describe alterations in brain functions related to mentalization and social cognition (Schurz et al., 2024). However, it is important to note that we did not assess the mentalization abilities of our participants. Given these limitations, as well as the modest significance of our findings, the implications of the mentalization network in subclinical participants warrant further investigation in future studies.

### 4.3 The central execution network

While the IC7 mostly overlaps with the DMN, some portions of the dlPFC and IFG, are usually ascribed to the CEN. If this is the case, we may conclude that some CEN alterations are only mildly visible in subclinical BPT. However, in our sample we could not find a separate CEN network to be associated with BPT. This could either be an indication of true absence in subclinical BPT, but it could also reflect methodical short comings, like ICA resolution, lack of power or statistical thresholding. Whether a functional involvement can be excluded in subclinical BPT deserves further exploration in future studies.

## 5 Conclusions and limitations

Our study presents a novel predictive model for borderline personality traits (BPT) through the analysis of resting-state fMRI data, highlighting distinct patterns of functional connectivity that may underlie the emotional and cognitive challenges associated with BPT. We identified two primary networks: increased BOLD temporal variability within the salience network, which correlates with heightened emotional sensitivity and reactivity, and decreased variability within a part of the default mode network, which may hinder the integration of complex social and self-referential information. These findings suggest that individuals with BPT, including those with subclinical presentations, exhibit a maladaptive emotional profile characterized by difficulties in emotion regulation and cognitive processing. The question remains as to whether functional changes or structural changes occur first and mediate alterations in the other. If we assume, the alterations start within the functional networks our results fit nicely with prior studies on BPT and BPD (e.g., Doll et al., 2013; Ruocco and Carcone, 2016; Langerbeck et al., 2023; Quattrini et al., 2022) suggesting a shift from subclinical to clinical presentation when structural alterations become visible. This view would support a partial divergence model between BPT and BPD, in which qualitative and quantitative differences are visible. This view and our results are consistent with the assumption that borderline personality is represented on a spectrum on which a shift in qualitative and quantitative changes is evident (e.g., from alterations in two functional and one structural network to alterations in all functional and all structural networks).

While this study offers valuable insights, there are some limitations. The first limitation relies on the use of a single psychometric tool to measure the Borderline trait. Future research could benefit from additional measures of BPT to possibly enhance consistency. Moreover, we focused on BOLD temporal variability, but other functional connectivity approaches, such as graph measures or ROI-to-ROI connectivity, could expand our understanding of the functional foundations of BPT. Incorporating self-representation and mentalization questionnaire could have helped capture the nuances of BPT profiles. In this study we could not find clear evidence for the CEN, indicating that the triple network model hypothesis deserves further exploration. Finally, longitudinal studies may reveal more about the stability of these BPT brain-behavior relationships over time. Last but not least, we used a decomposition of 20 ICs as suggested by the toolbox. This is in line with previous studies that have shown that using 20 ICs can effectively identify large-scale RS networks in an optimal way. Future studies may want to test a different number of decomposition.

## Data Availability

The original contributions presented in the study are included in the article/supplementary material, further inquiries can be directed to the corresponding author.

## References

[ref1] Aguilar-Ortiz S. Salgado-Pineda P. Vega D. Pascual J. C. Marco-Pallarés J. Soler J.. (2020). Evidence for default mode network dysfunction in borderline personality disorder. Psychol. Med. 50, 1746–1754. doi: 10.1017/S0033291719001880, PMID: 31456534

[ref2] American Psychiatric Association (2013). Diagnostic and Statistical Manual of Mental Disorders (DSM-5). 5th Edn. Washington, DC: American Psychiatric Association.

[ref3] Arioli M. Cattaneo Z. Ricciardi E. Canessa N. (2021). Overlapping and specific neural correlates for empathizing, affective mentalizing, and cognitive mentalizing: a coordinate-based meta-analytic study. Hum. Brain Mapp. 42, 4777–4804. doi: 10.1002/hbm.25570, PMID: 34322943 PMC8410528

[ref4] Babayan A. Baczkowski B. Cozatl R. Dreyer M. Engen H. Erbey M.. (2018). MPI-Leipzig mind-brain-body. OpenNeuro:ds000221. Available online at: https://openneuro.org/datasets/ds000221/versions/00002

[ref5] Baggio T. Grecucci A. Meconi F. Messina I. (2023). Anxious brains: a combined data fusion machine learning approach to predict trait anxiety from morphometric features. Sensors 23:610. doi: 10.3390/s23020610 36679404 PMC9863274

[ref6] Beeney J. E. Hallquist M. N. Ellison W. D. Levy K. N. (2016). Self–other disturbance in borderline personality disorder: neural, self-report, and performance-based evidence. Personal. Disord. Theory Res. Treat. 7:28. doi: 10.1037/per0000127, PMID: 26011577 PMC4659768

[ref7] Bozzatello P. Garbarini C. Rocca P. Bellino S. (2021). Borderline personality disorder: risk factors and early detection. Diagnostics 11:2142. doi: 10.3390/diagnostics11112142, PMID: 34829488 PMC8620075

[ref8] Buckner R. L. Carroll D. C. (2007). Self-projection and the brain. Trends Cogn. Sci. 11, 49–57. doi: 10.1016/j.tics.2006.11.004, PMID: 17188554

[ref9001] Calhoun V. D. Adali T. Pearlson G. D. Pekar J. J. (2001). A method for making group inferences from functional MRI data using independent component analysis. Hum Brain Mapp. 14, 140–51. doi: 10.1002/hbm.1048 11559959 PMC6871952

[ref9] Calhoun V. D. Miller R. Pearlson G. Adalı T. (2014). The chronnectome: time-varying connectivity networks as the next frontier in fMRI data discovery. Neuron 84, 262–274. doi: 10.1016/j.neuron.2014.10.015 25374354 PMC4372723

[ref10] Cavanna F. Vilas M. G. Palmucci M. Tagliazucchi E. (2018). Dynamic functional connectivity and brain metastability during altered states of consciousness. NeuroImage 180, 383–395. doi: 10.1016/j.neuroimage.2017.09.065 28986208

[ref11] Chang C. Glover G. H. (2009). Time-frequency dynamics of resting-state brain connectivity measured with FMRI. NeuroImage 50, 81–98. doi: 10.1016/j.neuroimage.2009.12.011, PMID: 20006716 PMC2827259

[ref12] Chen Y. Wang W. Zhao X. Sha M. Liu Y. Zhang X.. (2017). Age-related decline in the variation of dynamic functional connectivity: a resting state analysis. Front. Aging Neurosci. 9:203. doi: 10.3389/fnagi.2017.00203, PMID: 28713261 PMC5491557

[ref13] Chong J. S. X. Ng G. J. P. Lee S. C. Zhou J. (2017). Salience network connectivity in the insula is associated with individual differences in interoceptive accuracy. Brain Struct. Funct. 222, 1635–1644. doi: 10.1007/s00429-016-1297-7 27573028

[ref14] Costa P. T. McCrae R. R. (1992). Normal personality assessment in clinical practice: the NEO personality inventory. Psychol. Assess. 4:5. doi: 10.1037/1040-3590.4.1.5

[ref15] Cuthbert B. N. (2014). The RDoC framework: facilitating transition from ICD/DSM to dimensional approaches that integrate neuroscience and psychopathology. World Psychiatry 13, 28–35. doi: 10.1002/wps.20087, PMID: 24497240 PMC3918011

[ref16] Dadomo H. Grecucci A. Giardini I. Ugolini E. Carmelita A. Panzeri M. (2016). Schema therapy for emotional dysregulation: theoretical implication and clinical applications. Front. Psychol. 7:1987. doi: 10.3389/fpsyg.2016.01987, PMID: 28066304 PMC5177643

[ref17] De Panfilis C. Schito G. Generali I. Ossola P. Marchesi C. Grecucci A.. (2019). Emotions at the border: increased punishment behavior during fair interpersonal exchanges in borderline personality disorder. J. Abnorm. Psychol. 128, 162–172. doi: 10.1037/abn0000404 30714797

[ref18] Denny B. T. Fan J. Fels S. Galitzer H. Schiller D. Koenigsberg H. W. (2018). Sensitization of the neural salience network to repeated emotional stimuli following initial habituation in patients with borderline personality disorder. Am. J. Psychiatry 175, 657–664. doi: 10.1176/appi.ajp.2018.17030367, PMID: 29961363 PMC6032521

[ref19] Diveica V. Koldewyn K. Binney R. J. (2021). Establishing a role of the semantic control network in social cognitive processing: a meta-analysis of functional neuroimaging studies. NeuroImage 245:118702. doi: 10.1016/j.neuroimage.2021.118702, PMID: 34742940

[ref20] Doll A. Sorg C. Manoliu A. Wöller A. Meng C. Förstl H.. (2013). Shifted intrinsic connectivity of central executive and salience network in borderline personality disorder. Front. Hum. Neurosci. 7:727. doi: 10.3389/fnhum.2013.00727 24198777 PMC3812906

[ref21] Donegan N. H. Sanislow C. A. Blumberg H. P. Fulbright R. K. Lacadie C. Skudlarski P.. (2003). Amygdala hyperreactivity in borderline personality disorder: implications for emotional dysregulation. Biol. Psychiatry 54, 1284–1293. doi: 10.1016/s0006-3223(03)00636-x 14643096

[ref22] Fehlbaum L. V. Borbás R. Paul K. Eickhoff S. B. Raschle N. M. (2022). Early and late neural correlates of mentalizing: ALE meta-analyses in adults, children and adolescents. Soc. Cogn. Affect. Neurosci. 17, 351–366. doi: 10.1093/scan/nsab105, PMID: 34545389 PMC8972312

[ref23] Fonagy P. Luyten P. Strathearn L. (2011). Borderline personality disorder, mentalization, and the neurobiology of attachment. Infant Ment. Health J. 32, 47–69. doi: 10.1002/imhj.20283 28543560

[ref24] Ghomroudi P. A. Siugzdaite R. Messina I. Grecucci A. (2024). Resting-state fingerprints of acceptance and reappraisal. The role of sensorimotor, executive and affective networks. *arXiv*. Available online at: 10.48550/arXiv.2401.16533. [Epub ahead of preprint]

[ref25] Golec-Staśkiewicz K. Pluta A. Wojciechowski J. Okruszek Ł. Haman M. Wysocka J.. (2022). Does the TPJ fit it all? Representational similarity analysis of different forms of mentalizing. Soc. Neurosci. 17, 428–440. doi: 10.1080/17470919.2022.2138536, PMID: 36309870

[ref26] Goulden N. Khusnulina A. Davis N. J. Bracewell R. M. Bokde A. L. McNulty J. P.. (2014). The salience network is responsible for switching between the default mode network and the central executive network: replication from DCM. NeuroImage 99, 180–190. doi: 10.1016/j.neuroimage.2014.05.052 24862074

[ref27] Grecucci A. Dadomo H. Salvato G. Lapomarda G. Sorella S. Messina I. (2023). Abnormal brain circuits characterize borderline personality and mediate the relationship between childhood traumas and symptoms: a mCCA+jICA and random forest approach. Sensors 23:2862. doi: 10.3390/s23052862 36905064 PMC10006907

[ref28] Grecucci A. Lapomarda G. Messina I. Monachesi B. Sorella S. Siugzdaite R. (2022). Structural features related to affective instability correctly classify patients with borderline personality disorder. A supervised machine learning approach. Front. Psychiatry 13:804440. doi: 10.3389/fpsyt.2022.804440 35295769 PMC8918568

[ref29] Herpertz S. C. Schneider I. Schmahl C. Bertsch K. (2018). Neurobiological mechanisms mediating emotion dysregulation as targets of change in borderline personality disorder. Psychopathology 51, 96–104. doi: 10.1159/000488357 29672301

[ref30] Hopwood C. J. Zanarini M. C. (2010). Five-factor trait instability in borderline relative to other personality disorders. Persona. Disord. 1, 58–66. doi: 10.1037/a0018230, PMID: 22121460 PMC3222948

[ref31] Hörz-Sagstetter S. Ohse L. Kampe L. (2021). Three dimensional approaches to personality disorders: a review on personality functioning, personality structure, and personality organization. Curr. Psychiatry Rep. 23, 1–16. doi: 10.1007/s11920-021-01250-y PMC8238706 34181116

[ref32] Hung Y. Gaillard S. L. Yarmak P. Arsalidou M. (2018). Dissociations of cognitive inhibition, response inhibition, and emotional interference: Voxelwise ALE meta-analyses of fMRI studies. Hum. Brain Mapp. 39, 4065–4082. doi: 10.1002/hbm.24232 29923271 PMC6866358

[ref33] JASP Team. (2022). JASP (version 0.16.2). Available online at: https://jasp-stats.org/ (Accessed September, 2022).

[ref34] Krause-Utz A. Winter D. Niedtfeld I. Schmahl C. (2014). The latest neuroimaging findings in borderline personality disorder. Curr. Psychiatry Rep. 16:438. doi: 10.1007/s11920-014-0438-z 24492919

[ref35] Langerbeck M. Baggio T. Messina I. Bhat S. Grecucci A. (2023). Borderline shades: morphometric features predict borderline personality traits but not histrionic traits. NeuroImage Clin. 40:103530. doi: 10.1016/j.nicl.2023.103530 37879232 PMC10618757

[ref36] Li W. Yang P. Ngetich R. K. Zhang J. Jin Z. Li L. (2021). Differential involvement of frontoparietal network and insula cortex in emotion regulation. Neuropsychologia 161:107991. doi: 10.1016/j.neuropsychologia.2021.107991 34391808

[ref37] Long Y. Cao H. Yan C. Chen X. Li L. Castellanos F. X.. (2020). Altered resting-state dynamic functional brain networks in major depressive disorder: findings from the REST-meta-MDD consortium. NeuroImage Clin. 26:102163. doi: 10.1016/j.nicl.2020.102163 31953148 PMC7229351

[ref38] Long Y. Liu X. Liu Z. (2023a). Temporal stability of the dynamic resting-state functional brain network: current measures, clinical research Progress, and future perspectives. Brain Sci. 13:429. doi: 10.3390/brainsci13030429 36979239 PMC10046056

[ref39] Long Y. Ouyang X. Yan C. Wu Z. Huang X. Pu W.. (2023b). Evaluating test-retest reliability and sex-/age-related effects on temporal clustering coefficient of dynamic functional brain networks. Hum. Brain Mapp. 44, 2191–2208. doi: 10.1002/hbm.26202 36637216 PMC10028647

[ref40] Martin A. K. Dzafic I. Ramdave S. Meinzer M. (2017). Causal evidence for task-specific involvement of the dorsomedial prefrontal cortex in human social cognition. Soc. Cogn. Affect. Neurosci. 12, 1209–1218. doi: 10.1093/scan/nsx063 28444345 PMC5597860

[ref41] McCarthy-Jones S. Fernyhough C. (2011). The varieties of inner speech: links between quality of inner speech and psychopathological variables in a sample of young adults. Conscious. Cogn. 20, 1586–1593. doi: 10.1016/j.concog.2011.08.005 21880511

[ref42] Mendez-Miller M. Naccarato J. Radico J. A. (2022). Borderline personality disorder. Am. Fam. Physician 105, 156–161, PMID: 35166488

[ref43] Menon V. (2011). Large-scale brain networks and psychopathology: a unifying triple network model. Trends Cogn. Sci. 15, 483–506. doi: 10.1016/j.tics.2011.08.003 21908230

[ref44] Messina I. Bianco F. Cusinato M. Calvo V. Sambin M. (2016). Abnormal default system functioning in depression: implications for emotion regulation. Front. Psychol. 7:858. doi: 10.3389/fpsyg.2016.00858 27375536 PMC4901076

[ref45] Messina I. Bianco S. Sambin M. Viviani R. (2015). Executive and semantic processes in reappraisal of negative stimuli: insights from a meta-analysis of neuroimaging studies. Front. Psychol. 6:956. doi: 10.3389/fpsyg.2015.00956 26217277 PMC4499672

[ref46] Molenberghs P. Johnson H. Henry J. D. Mattingley J. B. (2016). Understanding the minds of others: a neuroimaging meta-analysis. Neurosci. Biobehav. Rev. 65, 276–291. doi: 10.1016/j.neubiorev.2016.03.020 27073047

[ref47] Morawetz C. Bode S. Derntl B. Heekeren H. R. (2017). The effect of strategies, goals and stimulus material on the neural mechanisms of emotion regulation: a meta-analysis of fMRI studies. Neurosci. Biobehav. Rev. 72, 111–128. doi: 10.1016/j.neubiorev.2016.11.014 27894828

[ref48] Omidvarnia A. Zalesky A. Mansour L. S. van de Ville D. Jackson G. D. Pedersen M. (2021). Temporal complexity of FMRI is reproducible and correlates with higher order cognition. NeuroImage 230:117760. doi: 10.1016/j.neuroimage.2021.117760 33486124

[ref49] Perez-Rodriguez M. M. Bulbena-Cabré A. Nia A. B. Zipursky G. Goodman M. New A. S. (2018). The neurobiology of borderline personality disorder. Psychiatr. Clin. North Am. 41, 633–650. doi: 10.1016/j.psc.2018.07.012 30447729

[ref50] Preti M. G. Bolton T. A. van de Ville D. (2017). The dynamic functional connectome: state-of-the-art and perspectives. NeuroImage 160, 41–54. doi: 10.1016/j.neuroimage.2016.12.061 28034766

[ref51] Puiu A. A. Wudarczyk O. Kohls G. Bzdok D. Herpertz-Dahlmann B. Konrad K. (2020). Meta-analytic evidence for a joint neural mechanism underlying response inhibition and state anger. Hum. Brain Mapp. 41, 3147–3160. doi: 10.1002/hbm.25004 32314475 PMC7336147

[ref52] Quattrini G. Magni L. R. Lanfredi M. Pedrini L. Carcione A. Riccardi I.. (2022). Aberrant structural connectivity of the triple network system in borderline personality disorder is associated with behavioral dysregulation. J. Clin. Med. 11:1757. doi: 10.3390/jcm11071757 35407365 PMC8999477

[ref53] Riedel M. C. Yanes J. A. Ray K. L. Eickhoff S. B. Fox P. T. Sutherland M. T.. (2018). Dissociable meta-analytic brain networks contribute to coordinated emotional processing. Hum. Brain Mapp. 39, 2514–2531. doi: 10.1002/hbm.24018 29484767 PMC5951754

[ref54] Ruocco A. C. Carcone D. (2016). A neurobiological model of borderline personality disorder: systematic and integrative review. Harv. Rev. Psychiatry 24, 311–329. doi: 10.1097/HRP.0000000000000123 27603741

[ref55] Scherpiet S. Brühl A. B. Opialla S. Roth L. Jäncke L. Herwig U. (2014). Altered emotion processing circuits during the anticipation of emotional stimuli in women with borderline personality disorder. Eur. Arch. Psychiatry Clin. Neurosci. 264, 45–60. doi: 10.1007/s00406-013-0444-x 24100929

[ref56] Schilbach L. Eickhoff S. B. Rotarska-Jagiela A. Fink G. R. Vogeley K. (2008). Minds at rest? Social cognition as the default mode of cognizing and its putative relationship to the “default system” of the brain. Conscious. Cogn. 17, 457–467. doi: 10.1016/j.concog.2008.03.013 18434197

[ref57] Schurz M. Berenz J. P. Maerz J. Perla R. Buchheim A. Labek K. (2024). Brain activation for social cognition and emotion processing tasks in borderline personality disorder: a meta-analysis of neuroimaging studies. Brain Sci. 14:395. doi: 10.3390/brainsci14040395 38672044 PMC11048542

[ref58] Schurz M. Radua J. Aichhorn M. Richlan F. Perner J. (2014). Fractionating theory of mind: a meta-analysis of functional brain imaging studies. Neurosci. Biobehav. Rev. 42, 9–34. doi: 10.1016/j.neubiorev.2014.01.009 24486722

[ref59] Schurz M. Radua J. Tholen M. G. Maliske L. Margulies D. S. Mars R. B.. (2021). Toward a hierarchical model of social cognition: a neuroimaging meta-analysis and integrative review of empathy and theory of mind. Psychol. Bull. 147, 293–327. doi: 10.1037/bul0000303 33151703

[ref60] Seeley W. W. (2019). The salience network: a neural system for perceiving and responding to homeostatic demands. J. Neurosci. 39, 9878–9882. doi: 10.1523/JNEUROSCI.1138-17.2019 31676604 PMC6978945

[ref61] Sharp C. Ha C. Carbone C. Kim S. Perry K. Williams L.. (2013). Hypermentalizing in adolescent inpatients: treatment effects and association with borderline traits. J. Personal. Disord. 27, 3–18. doi: 10.1521/pedi.2013.27.1.3 23342954

[ref62] Shunkai L. Chen P. Zhong S. Chen G. Zhang Y. Zhao H.. (2022). Alterations of insular dynamic functional connectivity and psychological characteristics in unmedicated bipolar depression patients with a recent suicide attempt. Psychol. Med. 53, 3837–3848. doi: 10.1017/S0033291722000484 35257645

[ref9002] Sorella S. Lapomarda G. Messina I. Frederickson J. J. Siugzdaite R. Job R.. (2019). Testing the expanded continuum hypothesis of schizophrenia and bipolar disorder. Neural and psychological evidence for shared and distinct mechanisms. Neuroimage Clin. 23:101854. doi: 10.1016/j.nicl.2019.101854 31121524 PMC6529770

[ref63] Spielberger C. D. (1996). State-trait anger expression inventory: professional manual. Lake Magdalene, FL: Psychological Assessment Resources, Inc.

[ref64] Tangney J. P. Baumeister R. F. Boone A. L. (2004). High self-control predicts good adjustment, less pathology, better grades, and interpersonal success. J. Pers. 72, 271–324. doi: 10.1111/j.0022-3506.2004.00263.x 15016066

[ref65] Taylor S. F. Liberzon I. (2007). Neural correlates of emotion regulation in psychopathology. Trends Cogn. Sci. 11, 413–418. doi: 10.1016/j.tics.2007.08.006 17928261

[ref66] Toller G. Brown J. Sollberger M. Shdo S. M. Bouvet L. Sukhanov P.. (2018). Individual differences in socioemotional sensitivity are an index of salience network function. Cortex 103, 211–223. doi: 10.1016/j.cortex.2018.02.012 29656245 PMC6143366

[ref67] Van Overwalle F. (2009). Social cognition and the brain: a meta-analysis. Hum. Brain Mapp. 30, 829–858. doi: 10.1016/j.euroneuro.2020.03.008 18381770 PMC6870808

[ref68] Viviani R. (2013). Emotion regulation, attention to emotion, and the ventral attentional network. Front. Hum. Neurosci. 7:746. doi: 10.3389/fnhum.2013.00746 24223546 PMC3819767

[ref69] Wittchen H. U. Kessler R. C. Zhao S. Abelson J. (1995). Reliability and clinical validity of UM-CIDI DSM-III-R generalized anxiety disorder. J. Psychiatr. Res. 29, 95–110. doi: 10.1016/0022-3956(94)00044-R 7666382

[ref70] Wright A. G. Hopwood C. J. Zanarini M. C. (2015). Associations between changes in normal personality traits and borderline personality disorder symptoms over 16 years. Personal. Disord. Theory Res. Treat. 6:1. doi: 10.1037/per0000092 PMC4293231 25364942

[ref71] Xiao Q. Shen L. He H. Wang X. Fu Y. Ding J.. (2024). Alteration of prefrontal cortex and its associations with emotional and cognitive dysfunctions in adolescent borderline personality disorder. Eur. Child Adolesc. Psychiatry 33, 3937–3949. doi: 10.1007/s00787-024-02438-2 38642117

[ref72] Zanella F. Monachesi B. Grecucci A. (2022). Resting-state BOLD temporal variability in sensorimotor and salience networks underlies trait emotional intelligence and explains differences in emotion regulation strategies. Sci. Rep. 12:15163. doi: 10.1038/s41598-022-19477-x 36071093 PMC9452559

[ref73] Zhang J. Cheng W. Liu Z. Zhang K. Lei X. Yao Y.. (2016). Neural, electrophysiological and anatomical basis of brain-network variability and its characteristic changes in mental disorders. Brain 139, 2307–2321. doi: 10.1093/brain/aww143 27421791

